# Removal of Discolouration of the Skin from Nitrate of Silver

**Published:** 1842-10-29

**Authors:** 


*Removal of Discolouration of the Skin from Nitrate of Silver.*—Dr. Patterson considers that "there can scarcely be a doubt, that in those cases, where the skin has become discoloured from the long use of nitrate of silver, the discolouration may be removed by the internal and external employment of suitable preparations of iodine."

The following is the formula which Dr. Patterson employs for the administration of the ioduret of silver.

℞. Iodureti Argenti,
Nitratis Potassæ, *aa* gr. x.,
Tere simul ut fiat pulvis subtil., dein adde
Pulv. Glycyrrhizæ ℥ss.
Sacchari Albi ℈j.
Mucil. Arab. q. s. M.
Fiant pil. xl. quarum sumat æger j. ter in die.

*Ibid.* Oct., 1842.

